# Supplementary material for: Cyclophilin BcCyp2 Regulates Infection-Related Development to Facilitate Virulence of the Gray Mold Fungus Botrytis cinerea
Source: Int J Mol Sci. 2021 Feb 8;22(4):1694. doi: 10.3390/ijms22041694 (PMC7914984; doi:10.3390/ijms22041694)
Supplement: Supplementary file 1 [file ijms-22-01694-s001.pdf]

Supporting Information

Cyclophilin BcCyp2 regulates infection-related development to facilitate virulence of the gray mold fungus *Botrytis cinerea*

Jiao Sun[1], Chen-Hao Sun[2], Hao-Wu Chang[3], Song Yang[2], Yue Liu[1], Ming-Zhe Zhang[2], Jie Hou[1, 4], Hao Zhang[3], Gui-Hua Li[2*] and Qing-Ming Qin[1*]

[1] College of Plant Sciences, Key Laboratory of Zoonosis Research, Ministry of Education, Jilin University, Changchun 130062, China

[2] College of Plant Sciences, Jilin University, Changchun 130062, China

[3] Key Laboratory of Symbolic Computation and Knowledge Engineering, Ministry of Education, College of Computer Science and Technology, Jilin University, Changchun 130012, China

[4] College of Forestry, Beihua University, Jilin 132013, China

[*]For correspondence:

E-mail: liguihua@jlu.edu.cn or qmqin@jlu.edu.cn

Competing interests: The authors declare that no competing interests exist.

Running title: BcCyp2 mediates fungal development and pathogenesis

The supplementary data contain seven Figures and two Tables.

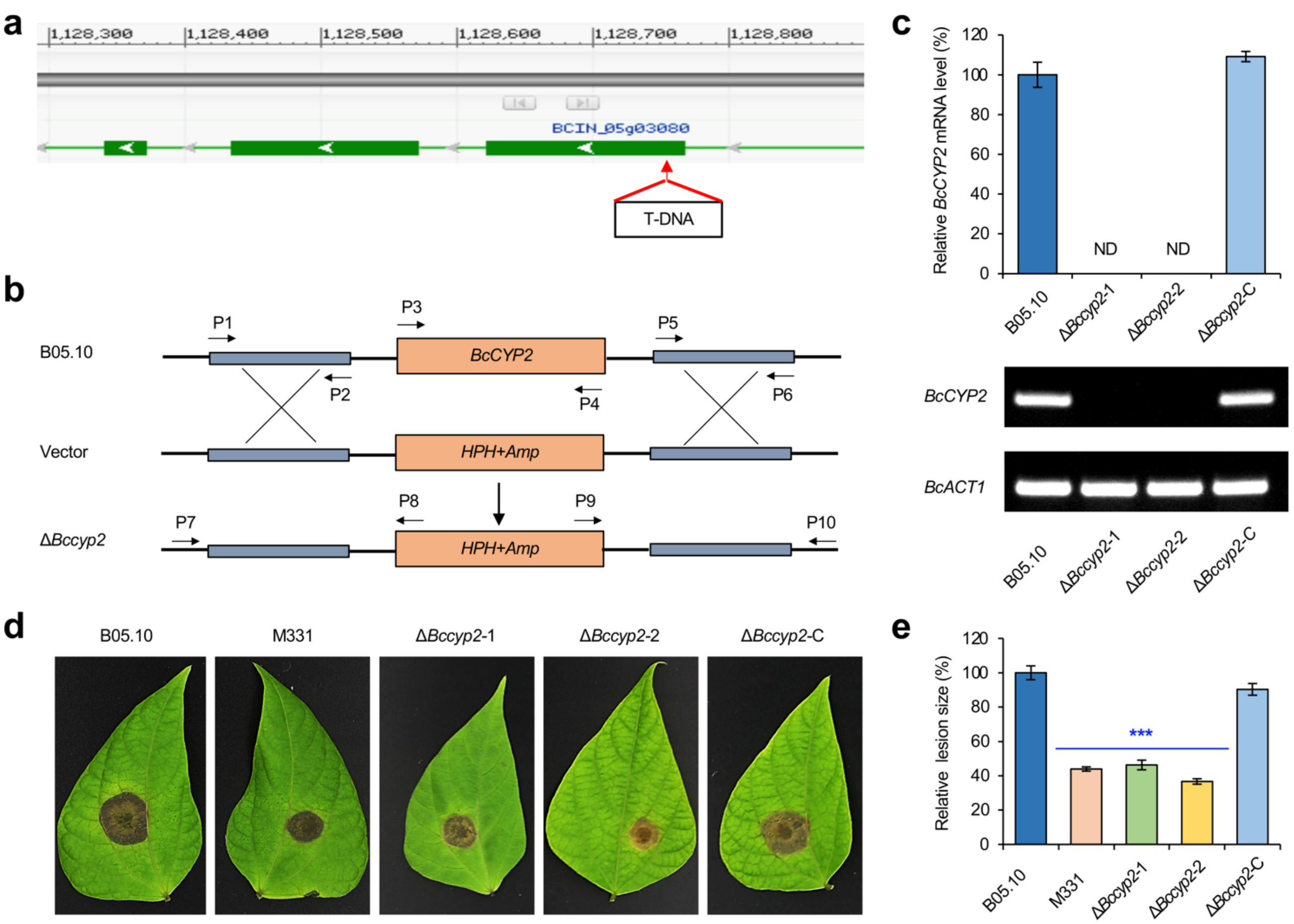

Figure S1. *Botrytis cinerea BcCYP2* is a virulence-associated gene. (a) A diagram showing T-DNA insertion site in the genome of the T-DNA insertional mutant M331. (b) Strategy for generation of *BcCYP2* disruption (Δ*Bccyp2*) strain. (c) Relative *BcCYP2* expression level in the indicated strains determined by quantitative reverse transcription PCR (qRT-PCR). (d-e) Disruption of *BcCYP2* in *B. cinerea* reduces the mutant virulence. Droplets of conidial suspension (1×10^5 conidia/mL, 10 µL) of the wild-type (WT) B05.10, T-DNA tagged mutant M331, Δ*Bccyp2*, and complemented strains were inoculated on intact green bean leaves and the inoculated leaves were photographically documented at 3 days post inoculation/incubation (dpi). ND: not detected. (d) and lesion sizes induced by the indicated strains were quantitatively analyzed (e). Data represent means ± standard deviations (SD) from at least three independent experiments. ***: significance at $p < 0.001$.

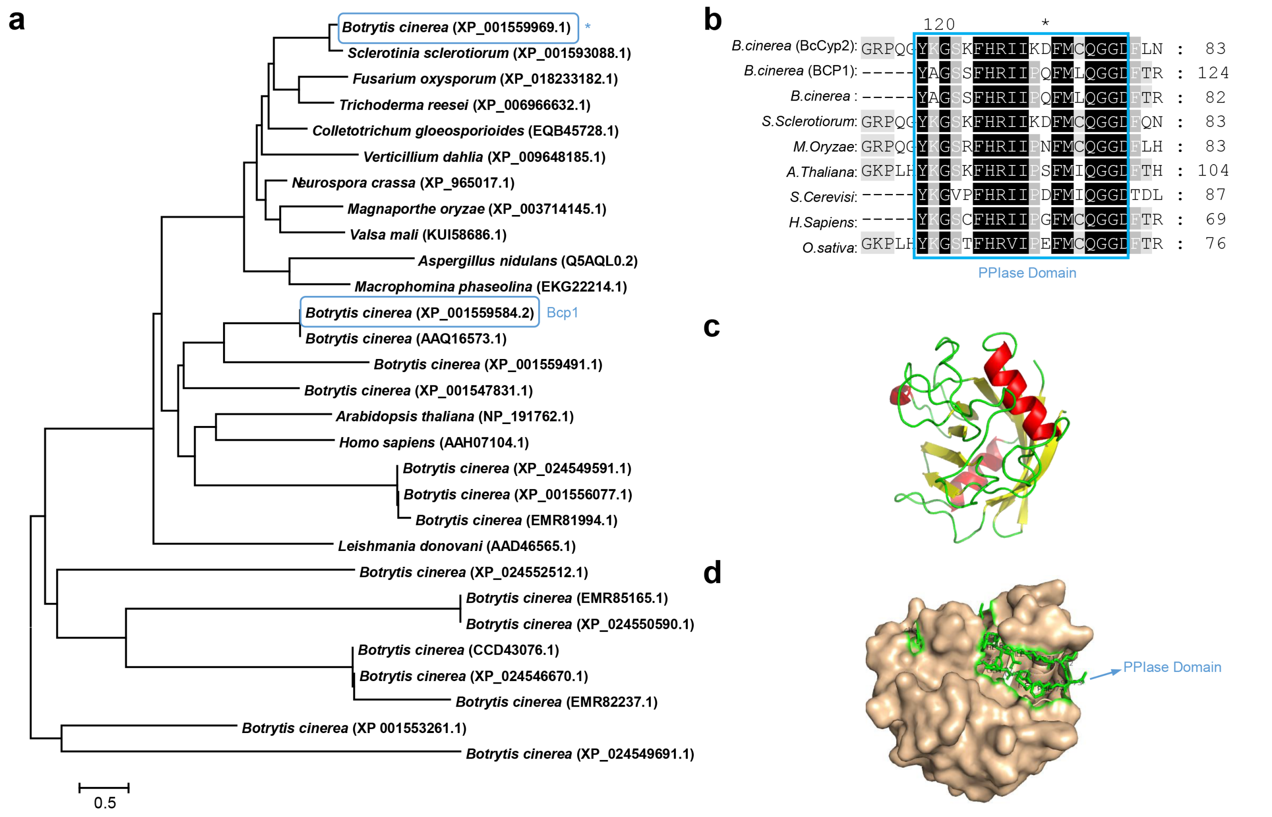

Figure S2. Sequence alignment and phylogenetic analyses of cyclophilin (Cyp) proteins from divergent organisms. (a) Phylogenetic analysis of Cyp proteins from the indicated organisms. Phylogenetic tree was generated using MEGA software (https://www.megasoftware.net/). (b) Sequence alignment of cyclophilin domains in Cyp proteins from the indicated organisms, including *B. cinerea* (XP_001559969.1)*, B. cinerea* (AAQ16572.1), *B. cinerea* (AAQ16573.1), *Sclerotinia sclerotiorum* (XP_001593088.1), *Magnaporthe oryzae* (XP_003714145.1), *Saccharomyces cerevisiae* (AJS85368.1), *Arabidopsis thaliana* (NP_191762.1), *Homo osapiens* (AAH07104.1), *Oryza sativa* (XP_015625227.1). Sequences were aligned using the GENEDOC (http://nrbsc.org/gfx/genedoc). (c) Three-dimensional structure of BcCyp2. The structure is predicted by SWISS-MODEL (https://swissmodel.expasy.org/interactive) and colored by PyMOL software (https://pymol.org/2/). (d): PPIase domain of BcCyp2. The green part indicates the predicted PPIase domain.

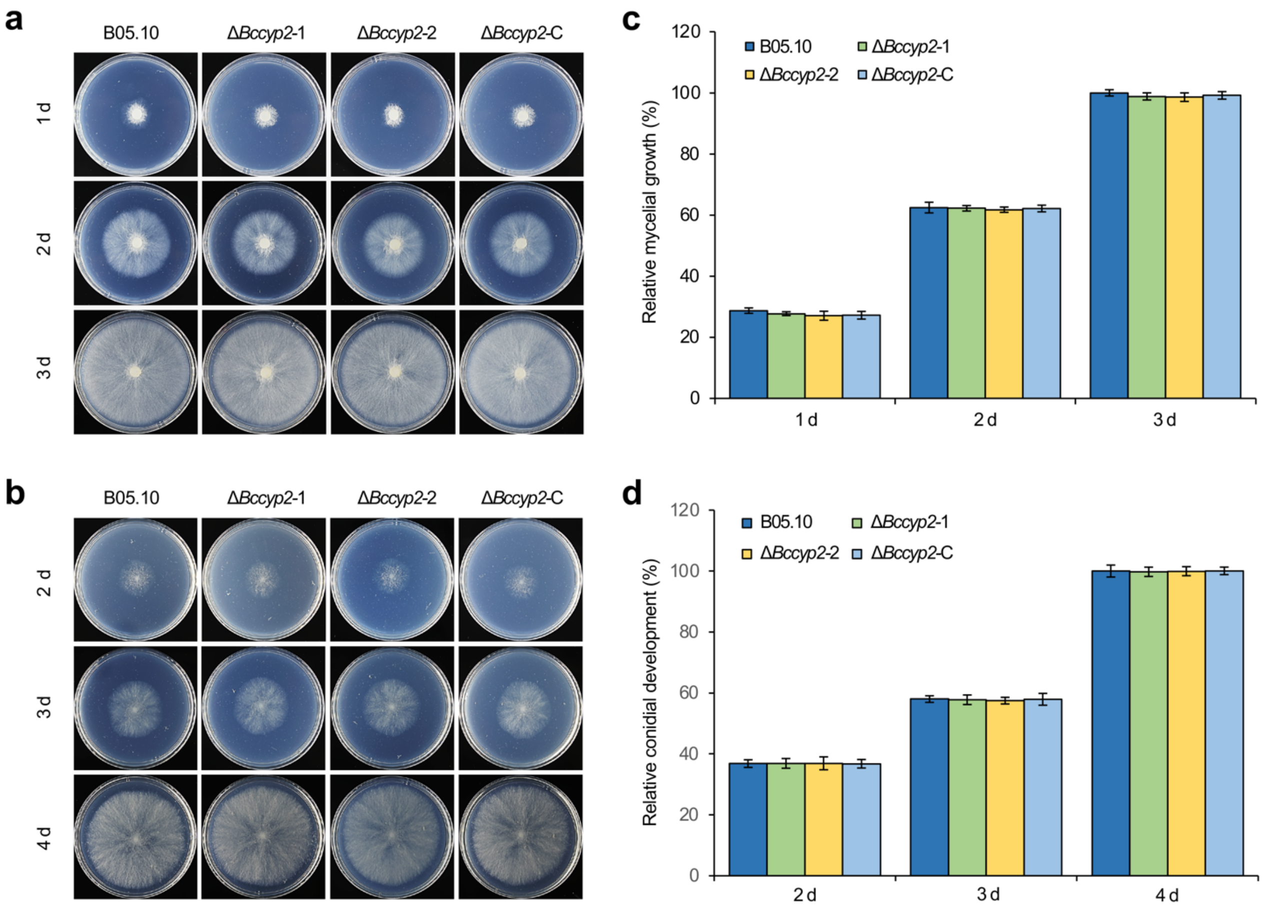

Figure S3. *BcCYP2* is dispensable for *B. cinerea* vegetative growth. (a-b) Radial growth of mycelia (a) or conidial development (b) of the indicated strains on complete medium (CM) plates at 20 °C in the dark. (c-d) Quantification of mycelial radical growth (c) or conidial development (d) of the indicated strains cultured on CM for 3 or 4 days, respectively. Representative images are from one of the experiments with triplicate plates examined for each strain in each experiment. Data represent means ± SD from at least three independent experiments.

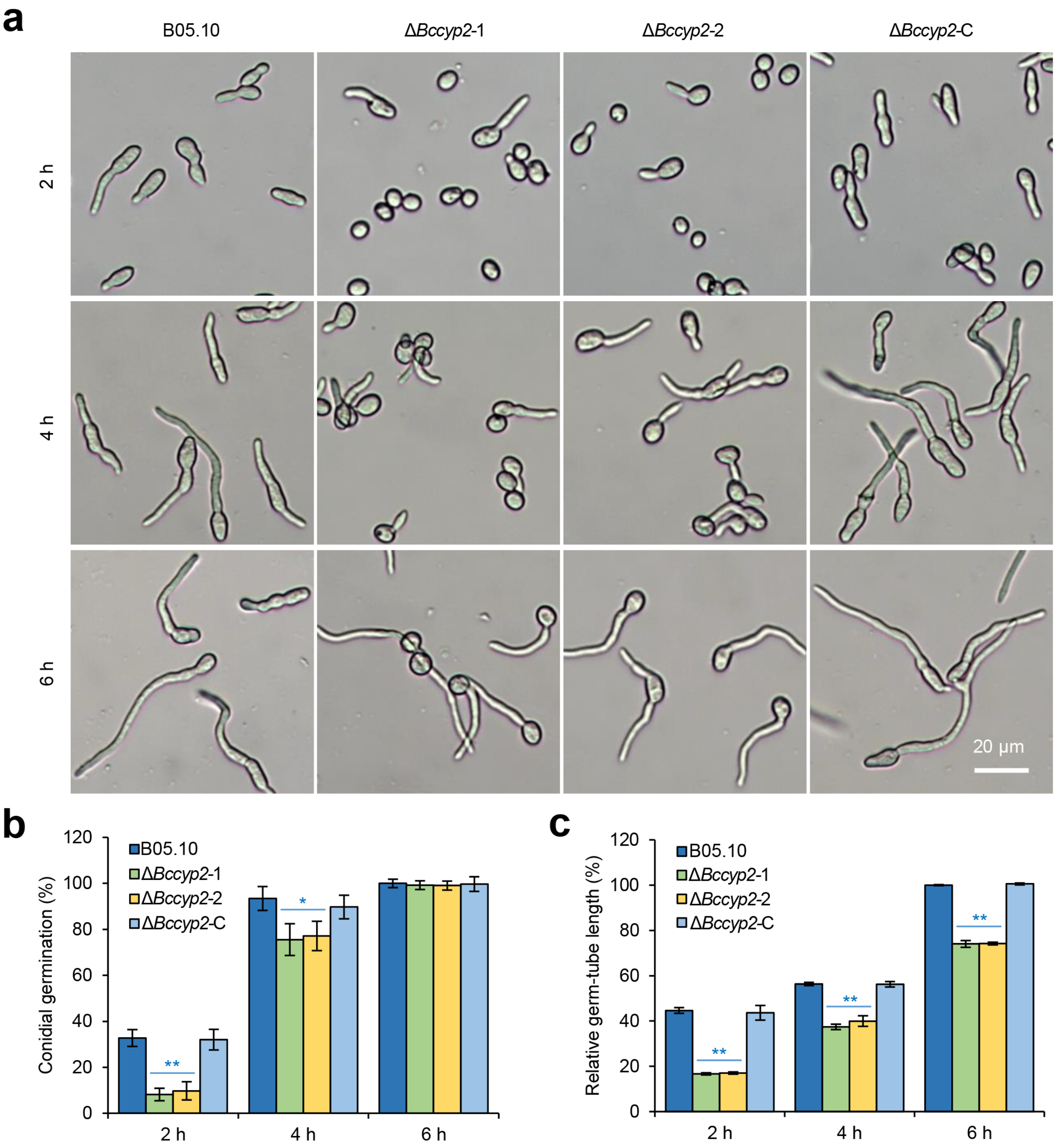

Figure S4. *BcCYP2* is required for *B. cinerea* conidial germination and germling development. (**a**) Conidial germination of the indicated strains. Conidia of each strain (1×10^6 conidia/mL) were mixed with liquid CM (vol: vol = 1: 1, 20 μL), inoculated on glass slides, and cultured at 20 °C in the dark. Conidial germination was photographically documented at 2, 4 and 6 hours post inoculation/incubation (hpi), respectively. (b, c) Quantitative analysis of conidial germination rate (b) or germ-tube development (c) of the tested strains. The representative images are from one of the experiments, at least three independent experiments were performed, and all the experiments resulted in similar results. Data represent means ± SD from at least four independent experiments. *, **: significance at $p < 0.05$ and 0.01, respectively.

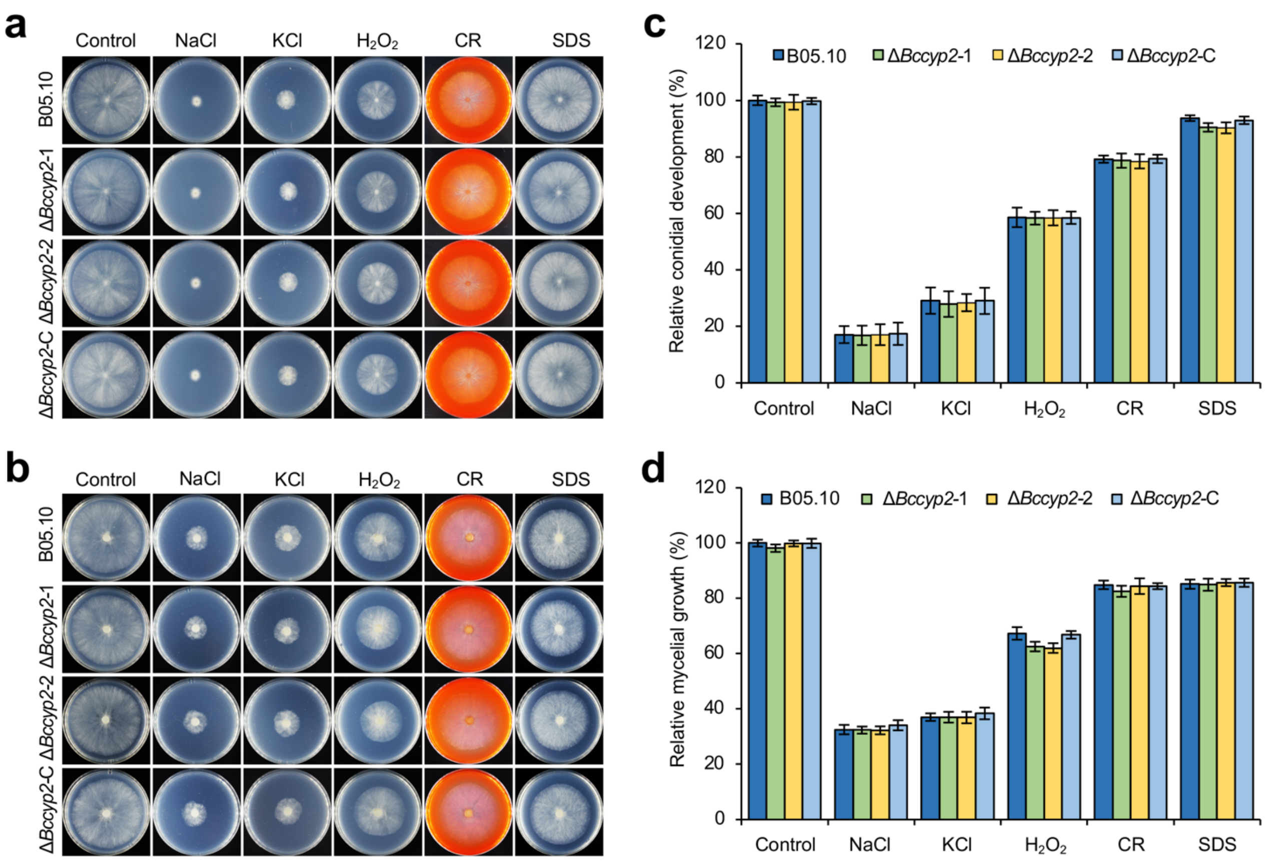

Figure S5. *BcCYP2* is dispensable for *B. cinerea* stress adaptation and cell wall integrity. (a) Growth of conidia (1×10^6 conidia/mL, 1 μL) of the indicated WT, Δ*Bccyp2*, and Δ*Bccyp2*-C strains of *B. cinerea* on CM plates containing the osmotic stress agents NaCl (1 M) and KCl (1 M), the oxidative-stress agent H_2O_2 (5 mM), or the cell wall disturbing reagents sodium dodecyl sulfate (SDS, 0.005%) and Congo Red (CR, 300 μg/mL). (b) Mycelial radial growth (inoculated with mycelial plugs from 3-day-old cultures) of the indicated strains on CM plates containing the indicated stress-mimetic agents as presented in (a). (c-d) Quantification of the relative mycelial growth of the indicated strains growing from inoculated conidia (c) or mycelial plugs (d) on CM plates supplemented with the indicated stress-mimetic agents. Representative images (at 4 dpi) are from one experiment. Data represent means ± SD from three independent experiments in which triplicate plates were examined for each strain in each experiment.

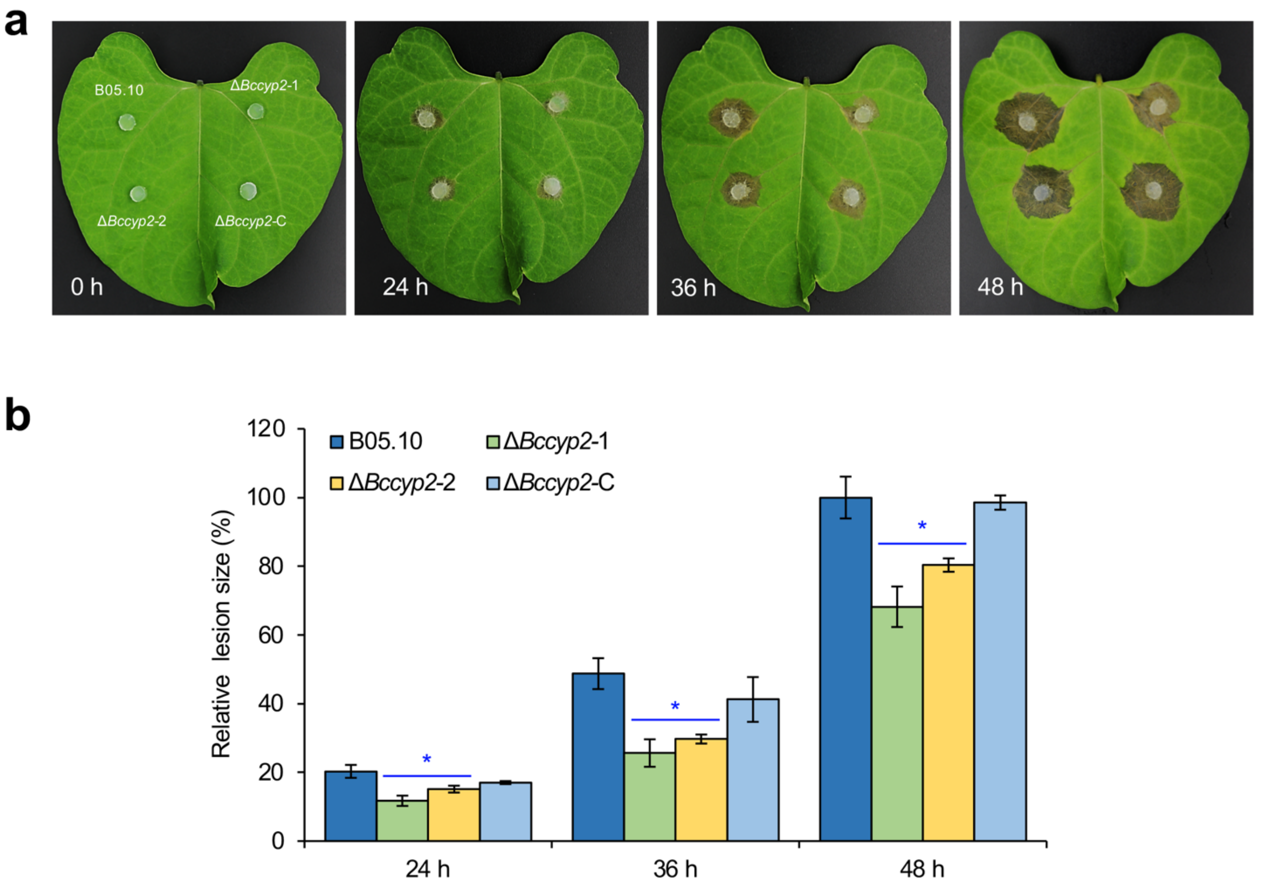

Figure S6. Loss of *BcCYP2* in *B. cinerea* impairs virulence of the pathogen. (a) Deletion of *BcCYP2* reduces the virulence of *B. cinerea*. Mycelial plugs of each strain were inoculated on green bean leaves and the lesions were photographically documented at the indicated hpi. (b) Quantification of lesion sizes caused by the indicated strains on unwounded green bean leaves shown in (a). Data represent means ± SD from three independent experiments. *: significance at $p < 0.05$.

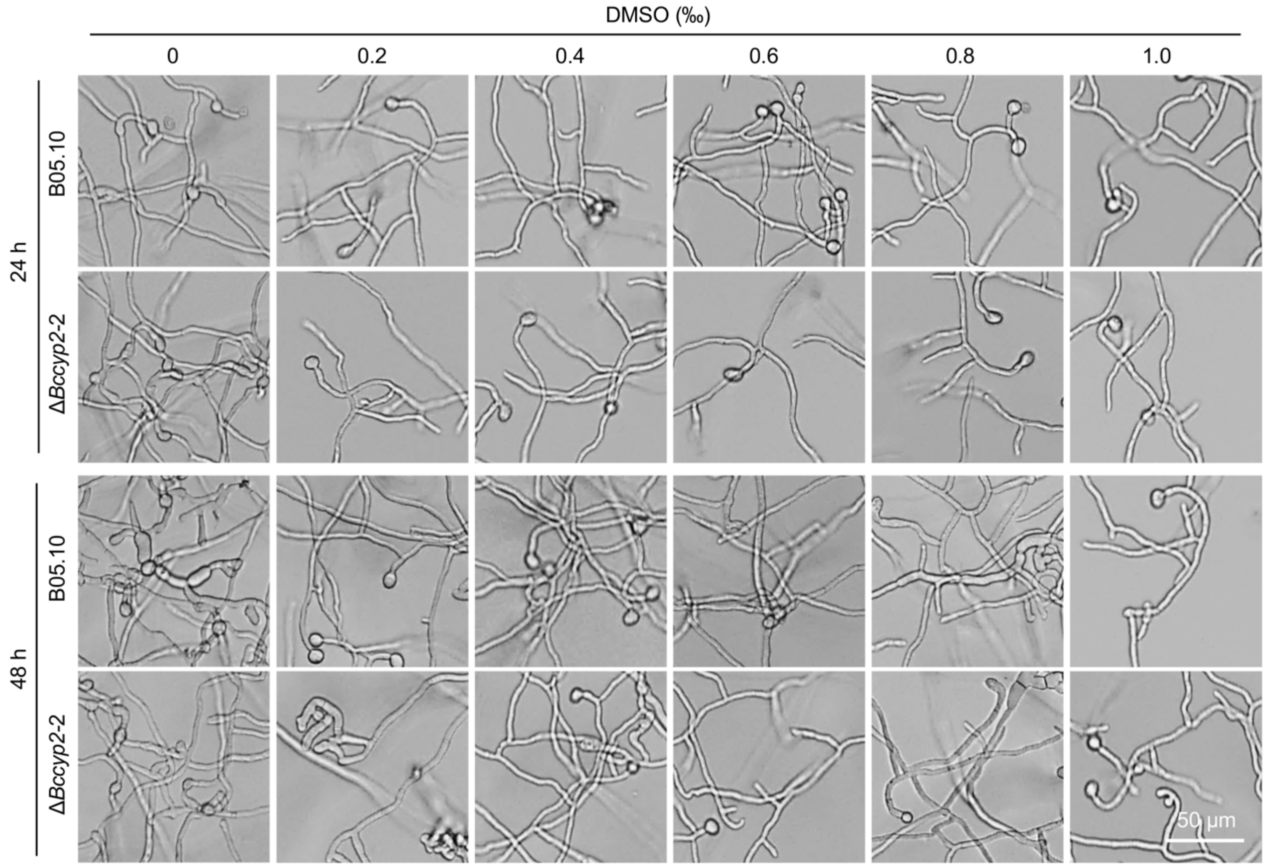

Figure S7. Dimethyl sulfoxide (DMSO) does not affect *B. cinerea* growth at the concentration that dissolved CsA. Conidia (5×10^4 conidia/mL in ½ liquid CM, 200 μL) of the indicated strains mixed with or without DMSO (0, 0.2, 0.4, 0.6, 0.8 or 1.0 ‰) were inoculated in 96-well plates and cultured at 20 °C. Conidial development and mycelial growth of the tested strains were observed and photographically documented at the indicated hpi.

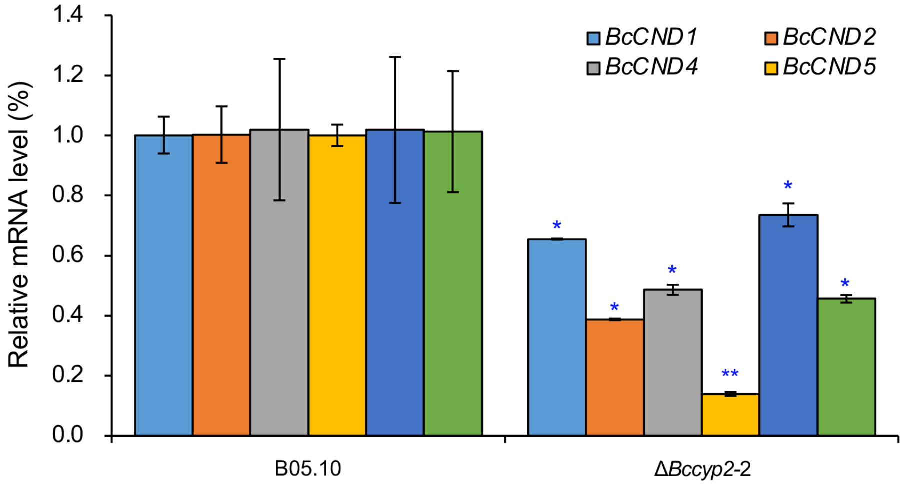

Figure S8. BcCyp2 regulates expression of calcineurin dependent (CND) genes. Conidia of the WT B05.10 or mutant Δ*Bccyp2*-2 strain were grown in liquid YG (yeast extract-glucose) medium for 60 h and then moved to the same medium without the nitrogen source (YG-N) and incubated for 4 h, the mycelia of the tested strains were further incubated with YG-N medium containing 0.5‱ DMSO (Vol: Vol) for 3 h, mycelia of the tested strains were then collected for RNA extraction. The mRNA levels of Ca^{2+}/calmodulin signaling genes were determined by qRT-PCR. Data represent means ± SD from three independent experiments. *, **: significance at $p < 0.05$ and 0.01, respectively.

Table S1. Subcellular localization and domains of putative cyclophilins in *B. cinerea.*

Protein	Subcellular localization		Domains
	BUSCA_fungi	Cell-Ploc (Euk-mPLoc 2.0)	NCBI
AAQ16573.1	extracellular space	Mitochondrion. Nucleus	cyclophilin_ABH_like
CCD43076.1	cytoplasm	Cytoplasm	cyclophilin_WD40
EMR81994.1	endomembrane system	Endoplasmic reticulum. Golgi apparatus. Mitochondrion. Nucleus	cyclophilin_ABH_like
EMR82237.1	cytoplasm	Cytoplasm	cyclophilin_WD40
EMR85165.1	cytoplasm	Nucleus	cyclophilin_RING
XP_001547831.1	cytoplasm	Endoplasmic reticulum	cyclophilin_ABH_like
XP_001553261.1	extracellular space	Nucleus	cyclophilin_SpCYP2_like
XP_001556077.1	endomembrane system	Endoplasmic reticulum. Golgi apparatus. Mitochondrion. Nucleus.	cyclophilin_ABH_like
XP_001559491.1 (Bcp1)	mitochondrion	Mitochondrion. Nucleus	cyclophilin_ABH_like
XP_001559584.2	mitochondrion	Mitochondrion. Nucleus	cyclophilin_ABH_like
XP_001559969.1 (BcCyp2)	extracellular space	Nucleus	cyclophilin_ABH_like
XP_024546670.1	cytoplasm	Cytoplasm	cyclophilin_WD40
XP_024549591.1	endomembrane system	Endoplasmic reticulum. Golgi apparatus. Mitochondrion.	cyclophilin_ABH_like
XP_024549691.1	nucleus	Cytoplasm. Nucleus	cyclophilin_CeCYP16_like
XP_024550590.1	cytoplasm	Nucleus	cyclophilin_RING
XP_024552512.1	mitochondrion	Cytoplasm	cyclophilin_PPIL3_like

Table S2. Primers used in this study.

Primer	Sequence (5'→3')	Purpose	References
BCRB-1	GGCACTGGCCGTCGTTTTACAAC	Right border amplification of T-DNA in TAIL-PCR	This study
BCRB-2	AACGTCGTGACTGGGAAAACCCT	Right border amplification of T-DNA in TAIL-PCR	This study
BCRB-3	CCCTTCCCAACAGTTGCGCA	Right border amplification of T-DNA in TAIL-PCR	This study
BCLB-1	GGGTTCCTATAGGGTTTCGCTCATG	Left border amplification of T-DNA in TAIL-PCR	This study
BCLB-2	CATGTGTTGAGCATATAAGAAACCCT	Left border amplification of T-DNA in TAIL-PCR	This study
BCLB-3	GAATTAATTCGGCGTTAATTCAGT	Left border amplification of T-DNA in TAIL-PCR	This study
BCAD1	TGWGNAGWANCASAGA	Degenerate primer in TAIL-PCR	This study
BCAD2	WAGTGNAGWANCANAGA	Degenerate primer in TAIL-PCR	This study
BCAD3	STTGNTASTNCTNTGC	Degenerate primer in TAIL-PCR	This study
BCAD4	WCAGNTGWTNGTNCTG	Degenerate primer in TAIL-PCR	This study
P1	GATCTTCACTAGTGGGAATTCCTCCGTATTGCTCTTTCCG	Amplification of 5' flank for *BcCYP2*	This study
P2	TTGGGTACCGAGCTCGAATTCAACTCCGATTGTCCTCCTG	Amplification of 5' flank for *BcCYP2*	This study
P5	AAAGATCAAAGGATCGTCGACTGATTTCTTGAATGGTGATGGCACG	Amplification of 3' flank for *BcCYP2*	This study
P6	CTTGCATGCCTGCAGGTCGACGGATTGTCTATCTGATCCTCC	Amplification of 3' flank for *BcCYP2*	This study
P3	AGCAGCGGAAAGAAGGTCA	Diagnostic PCR – homologous integration at 5'	This study
P4	AACTACATCGGCGAAGAGC	Diagnostic PCR – homologous integration at 3'	This study
HpTb	TGAACTCACCGCGACGTCTGT	Diagnostic PCR for *HPH*	(Zhang et al., 2020)
HpTa	TGCGCCCAAGCTGCATCAT	Diagnostic PCR for *HPH*	(Zhang et al., 2020)
P8	AATCGCAGCGTGAGGTGT	Diagnostic PCR – homologous integration at 5' (*HPH*)	This study
P9	CGGGATTTGATTAAAGATTTCTC	Diagnostic PCR – homologous integration at 3' (*HPH*)	This study
BcCYP2-CF (P7)	CTCCACCGCGGTGGCGGCCGCTCTAGACTGCCTCCATCACATCCTT	Amplification of *BcCYP2* complemented sequence (Diagnostic PCR - homologous integration at 5')	This study
BcCYP2-CR (P10)	CAGCCCGGGGGATCCACTAGTTCTAGAATCATTTCACTCCAACTCCTACA	Amplification of *BcCYP2* complemented sequence (Diagnostic PCR - homologous integration at 3')	This study
BcCYP2-1	AGGTGAGCCACTCGGTCG	qRT-PCR for *BcCYP2*	This study

Primer	Sequence (5'→3')	Purpose	References
BcCYP2-2	CCTTTGTATCCTTGCGGTCT	qRT-PCR for *BcCYP2*	This study
BcActin-F	CATGGCTGGTCGTGATTTGA	qRT-PCR for *BcACT1*	(Liu et al., 2018)
BcActin-R	GAGGATTGACTGGCGGTTTG	qRT-PCR for *BcACT1*	(Liu et al., 2018)
qBcCND1-F	CGCCGCTATCATCCCTCT	qRT-PCR for *BcCND1*	This study
qBcCND1-R	CGGTAGCGGCGTTCTTG	qRT-PCR for *BcCND1*	This study
qBcCND2-F	GCCGCTTCCAAGTCCAA	qRT-PCR for *BcCND2*	This study
qBcCND2-R	GGTTCCCTTTCCATCCCAGT	qRT-PCR for *BcCND2*	This study
qBcCND4-F	ATTAGCGGCTCCACGACTC	qRT-PCR for *BcCND4*	This study
qBcCND4-R	AAGCCTTATGCCCTCCACC	qRT-PCR for *BcCND4*	This study
qBcCND5-F	CCCAAGAACGACCAAGGC	qRT-PCR for *BcCND5*	This study
qBcCND5-R	ACCGCTCGGGGATGAAC	qRT-PCR for *BcCND5*	This study
qBcCND8-F	CGCTGCGAAACGCTACATT	qRT-PCR for *BcCND8*	This study
qBcCND8-R	TAAAGTTATCACTCATCTCCACCAC	qRT-PCR for *BcCND8*	This study
qBcCND10-F	TGCCCCTCTATCCCTACGG	qRT-PCR for *BcCND10*	This study
qBcCND10-R	ATCTCCCCTTCCCTGTCTGT	qRT-PCR for *BcCND10*	This study

References

Liu, J.K., Chang, H.W., Liu, Y., Qin, Y., Ding, Y.H., Wang, L., Zhao, Y., Zhang, M.Z., Cao, S.N., and Li, L.T. (2018). The key gluconeogenic gene PCK1 is crucial for virulence of Botrytis cinerea via initiating its conidial germination and host penetration. Environmental microbiology *20*, 1794-1814.

Zhang, M.Z., Sun, C.H., Liu, Y., Feng, H.Q., Chang, H.W., Cao, S.N., Li, G.H., Yang, S., Hou, J., Zhu-Salzman, K., *et al.* (2020). Transcriptome analysis and functional validation reveal a novel gene, BcCGF1, that enhances fungal virulence by promoting infection-related development and host penetration. Molecular Plant Pathology *21*, 834–853.
